# Comparison of Intravenous Granisetron and Ondansetron on Hemodynamics and Sensory Motor Block After Spinal Anaesthesia With Hyperbaric Bupivacaine in Patients Undergoing Elective Surgery: A Randomized Double-Blind Study

**DOI:** 10.7759/cureus.36383

**Published:** 2023-03-20

**Authors:** Usha Shukla, Manoj Kumar, Kapil K Gautam, Jay Brijesh Singh Yadav

**Affiliations:** 1 Anaesthesiology and Critical Care, Uttar Pradesh University of Medical Sciences, Etawah, IND; 2 Anaesthesiology, Uttar Pradesh University of Medical Sciences, Etawah, IND

**Keywords:** granisetron, bupivacaine, sensory motor block, hemodynamics, ondansetron

## Abstract

Background: Bezold Jarisch reflex (BJR) is mediated by peripheral serotonin receptor 5-HT_3_ type. BJR and sympathetic blockade are important causes of hypotension and bradycardia after spinal anaesthesia. Premedication with serotonin receptor antagonists has a role in the attenuation of hemodynamic disturbances.

Aim: To compare the effect of intravenous granisetron and ondansetron on the hemodynamic and sensory-motor block after spinal anaesthesia with hyperbaric bupivacaine in patients undergoing elective surgery.

Methodology: Ninety patients posted for elective surgery under spinal anaesthesia were randomly divided into three groups of 30 each. Group A patients received ondansetron 4mg, group B received granisetron 1mg, and group C received normal saline intravenously. Hemodynamic variables such as heart rate, systolic blood pressure, diastolic blood pressure, mean arterial pressure, and peripheral oxygen saturation, were recorded at baseline and then 2 minutes intervals for 20 minutes and thereafter every 5 minutes till the end of the surgery. The onset and duration of sensory and motor block were recorded at baseline and then every 2 minutes till the complete block was achieved.

Result: No patient was excluded from our study. During the intergroup comparison, heart rate and mean arterial pressure remained stable in group A compared to groups B and C. Time to reach peak sensory block level T4 was faster in group A compared to group B and group C. The rate of sensory block regression to two segments (T4 to T6) and thereafter up to T10, T12, and S1 was faster in group B compared to groups A and C. The attainment of complete motor block, Modified Bromage Score (MBS)=4 was faster in group A compared to group B and group C. The rate of motor block regression to MBS=3 and MBS=0 was faster in group B compared to group A and group C.

Conclusion: Premedication with ondansetron 4mg and granisetron 1mg intravenously significantly reduces ephedrine use. Ondansetron provides better hemodynamic stability, earlier onset of the sensory and motor blocks as well as prolonged duration of sensory and motor blocks, and duration of analgesia compared to granisetron.

## Introduction

Spinal anaesthesia is the most commonly performed procedure in obstetrics patients for caesarean section [1]. The preferred modality of anaesthesia for surgery related to the lower abdominal and lower extremities is spinal anaesthesia over general anaesthesia because it provides postoperative analgesia, decreased blood loss, and fewer adverse effects. Complications like bradycardia, hypotension, shivering, nausea and vomiting, failure of the block, pain during injection, headache, and backache are commonly associated with subarachnoid block [2]. The mechanism involved in hypotension is a decrease in systemic vascular resistance due to sympathetic blockade. This subsequently results in blood pooling as a result of vasodilatation and finally decreases in preload leading to falling in blood pressure. This decrease in preload stimulates serotonin-sensitive chemoreceptors and mechanoreceptors in the ventricular wall [3]. Serotonin (5-HT3 subtype) receptors are located centrally in the chemoreceptor trigger zone and peripherally as cardiac chemoreceptors on the cardiac vagal afferent [4]. Stimulation of cardiac mechanoreceptors activates the Bezold-Jarisch reflex mechanism, causing inhibition of the vasomotor centre which promotes hypotension as a result of vasodilatation and increases the parasympathetic activity leading to bradycardia [5].

Hemodynamic changes can be antagonized by premedication with selective 5-HT3 receptor antagonists [6]. Ondansetron is a potent, selective, competitive antagonist at 5-HT3 receptors, which have a role in the attenuation of spinal anaesthesia-induced episodes of nausea, vomiting, and hypotension [7]. However lower doses of ondansetron were preferred as the risk of prolonged Q-T interval was more with a higher dose, while the efficacy remains constant [8]. Granisetron is a highly selective 5-HT3 receptor antagonist and effectively attenuates spinal anaesthesia-induced hypotension [9]. This potent 5-HT3 receptor antagonist can be safely administered at a dosage of 1mg to 3mg, with a dose-dependent increase in efficacy, with minimal to no risk of cardiac adverse effects [10]. Pre-treatment with ondansetron and granisetron enhances regression of sensory-motor block after intrathecal administration of 5% hyperbaric lignocaine and hyperbaric bupivacaine respectively [11].

Though so many studies are available in favour of the beneficial effects of ondansetron for preventing spinal anaesthesia-induced hypotension and bradycardia and its effect on sensory-motor blockade duration but much work is not done on granisetron individually and very few studies of the two drugs with low doses are available. Therefore, we planned a prospective randomized double-blind study to evaluate and compare the effects of premedication with low dose ondansetron and granisetron on hemodynamic changes, sensory and motor block after spinal anaesthesia with 0.5% hyperbaric bupivacaine administered intrathecally.

## Materials and methods

This prospective randomized, double‑blinded study was conducted after obtaining approval from the Institutional Ethical Committee of Uttar Pradesh University of Medical Sciences, Saifai, Etawah (Ref. No.: 1885/UPUMS/Dean(M)/Ethical/2020-21E.C.153/2020-21). Informed and written consent was taken from patients.

Inclusion and exclusion criteria

Patients aged 18-65 years, of either sex, American Society of Anaesthesiologists physical status classification I and II posted for elective surgery under spinal anaesthesia were included in the study. Patients with known hypersensitivity to study drugs, peripheral vascular disease, ischemic heart disease, having a cardiac pacemaker, contraindication to subarachnoid block, and taking any heart rate modifying drugs were excluded from the study.

Sample size calculation and randomization

Sample size calculation was done based on 80% power, 5% significance level with a 95% confidence interval as well as absolute error being 0.10 and assumed standard deviation being 31.9 and 21.4, the total sample size calculated was 90 patients divided into 30 patients per group. Patients were randomly allocated using sequentially numbered cards in sealed opaque envelopes to one of the following groups:

Group A received ondansetron 4mg diluted in normal saline,

Group B received granisetron 1mg diluted in normal saline,

Group C received normal saline.

The total volume of the solution infused was kept at 10 mL.

All the patients underwent detailed pre-anaesthetic evaluation. All the patients received a tablet of alprazolam 0.25mg and a tablet of pantoprazole 40mg orally at night before surgery. Patients were kept nil per oral for 6-8 hours before surgery. In the preoperative room, peripheral vascular access was obtained by an 18-gauge cannula in all the patients and preloading was done with lactated ringer’s solution at 20mL/kg of body weight over 20 minutes. In the operation room, standard monitors including electrocardiography, non-invasive blood pressure, and pulse oximeter were attached and hemodynamic parameters like heart rate (HR), systolic blood pressure (SBP), diastolic blood pressure (DBP), mean arterial pressure (MAP), electrocardiograph (ECG), peripheral oxygen saturation (SPO2) were recorded. After preloading, patients in the respective group received the infusion of the study drug over 1 minute just 5 minutes before performing the subarachnoid block.

Procedure

After all aseptic precautions, a subarachnoid block was performed at L3-L4 intervertebral space using a 25-gauge Quincke’s spinal needle. 0.5% hyperbaric bupivacaine 2.0mL was administered intrathecally slowly after a free flow of cerebrospinal fluid was seen.

Hemodynamic variables like HR, SBP, DBP, MAP, and SPO2 were recorded at baseline and then 2 minutes intervals for 20 minutes and thereafter every 5 minutes till the end of the surgery. All the data was recorded by an anaesthesiologist who was blinded to the study group.

The sensory block level was assessed by pinprick method every 2 minutes along the mid-clavicular line bilaterally from caudal to rostral direction till peak sensory block level T4 was achieved and then after every 10 minutes till complete sensory block regression, according to Gromley and Hill grade. The motor block level was assessed by the Modified Bromage Scale (MBS) every 2 minutes till the complete motor block was achieved then after every 15 minutes till complete motor recovery.

Statistical methods

All the recorded data was summarized, tabulated, and statistically analysed using the Statistical Package of Social Science (SPSS) 20.0 (IBM Corp., Armonk, NY, U.S.A.). The quantitative data were summarized as mean ± standard deviation and analysed by using ANOVA test among the groups and using unpaired t-test between the groups. Qualitative variables were compared using the Chi-square test. P value <0.05 is considered statistically significant and highly significant if P value<0.001.

## Results

**Figure 1:**
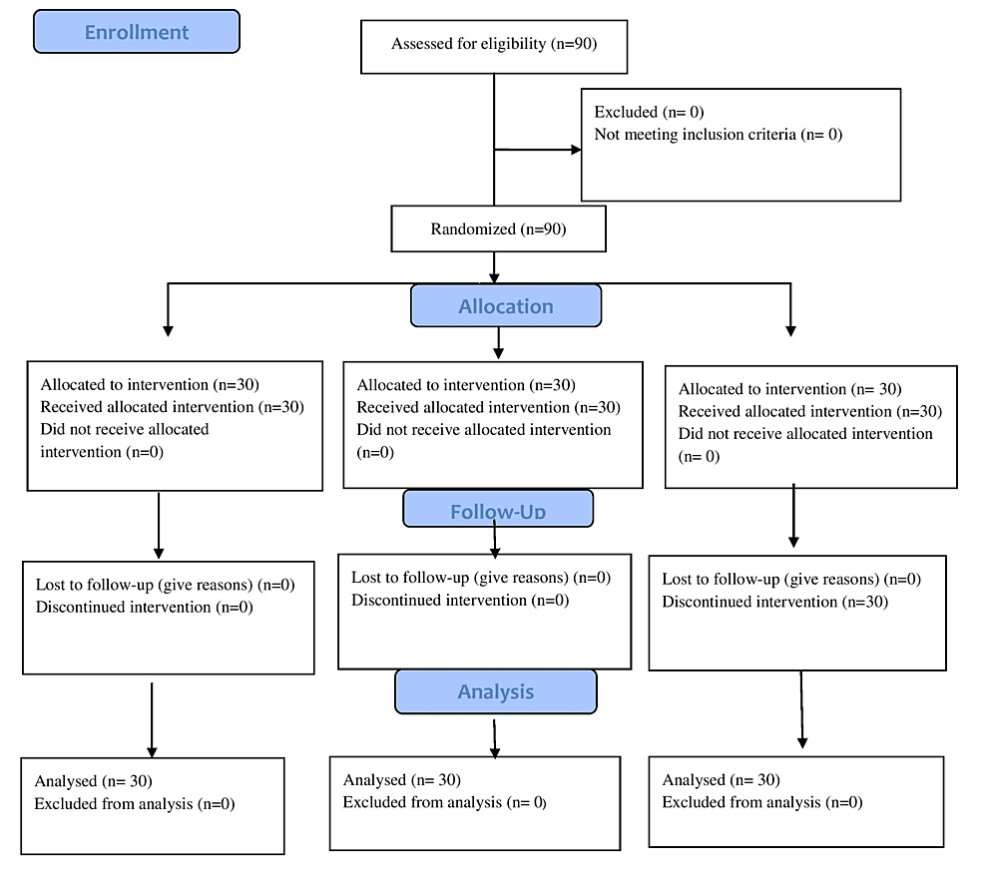
CONSORT flow diagram

A total of 90 patients were enrolled in the study and divided into three groups of 30 each. No patients were excluded from our study as shown in the consort chart (Figure 1).

All the groups were comparable in terms of using demographic data, anthropometric parameters, duration of surgery, and American Society of Anesthesiologists (ASA) physical status (Table 1).

**Table 1:** Demographic characteristics and operative data BMI: body mass index; ASA-PS: American Society of Anesthesiologists-Physical Status

Characteristics	Group A (n=30) Mean±SD	Group B (n=30) Mean±SD	Group C (n=30) Mean±SD	P-value
Age (years)	42.10±11.26	39.57±14.11	38.40±13.66	0.536
Height (centimetres)	164.50±6.86	165.63±5.6	164.13±6.2	0.626
Weight (kilograms)	60.90±8.69	60.77±7.2	61.27±6.11	0.964
BMI (kg/m^2^)	22.41±1.99	22.10±1.95	22.76±2.14	0.456
Duration of Surgery (minutes)	53.40±14.05	55.63±10.85	52.37±11.29	0.569
ASA-PS (1:2)	26:4	18:12	22:8	0.065
Sex % (male: female)	83.33:16.67	83.33:16.67	63.33:36.67	0.143

Hemodynamic parameters

The heart rate was comparable among the groups at all time periods during the surgery (P-value>0.05) (Table 2).

**Table 2:** Comparison of mean heart rate (beats per minute) among the study groups

Time (minutes)	Group A (n=30)	Group B (n=30)	Group C (n=30)	P value	P value		
	Mean±SD	Mean±SD	Mean±SD		A vs B	A vs C	B vs C
0 min	78.63±4.62	79.13±4.25	78.83±4.38	0.907	0.332	0.432	0.394
2 min	78.67±4.05	77.73±4.05	78.50±3.25	0.596	0.188	0.431	0.211
4 min	78.30±3.82	77.87±5.11	76.83±4.91	0.456	0.356	0.101	0.214
6 min	78.00±4.83	77.57±5.76	76.30±6.34	0.486	0.377	0.124	0.211
8 min	77.73±4.19	76.10±8.64	76.00±6.41	0.531	0.178	0.110	0.480
10 min	78.10±5.04	77.27±8.65	77.63±7.52	0.905	0.325	0.389	0.431
12 min	79.00±5.19	78.10±8.4	77.83±7.15	0.797	0.310	0.236	0.448
14 min	79.27±4.91	79.90±10.15	79.37±6.24	0.940	0.380	0.473	0.404
16 min	78.23±4.88	79.93±8.01	79.20±9.48	0.693	0.162	0.311	0.374
18 min	78.60±4.32	78.57±8.1	79.13±4.99	0.922	0.492	0.330	0.373
20 min	79.20±4.74	80.20±8.13	79.20±7.85	0.819	0.281	0.500	0.315
25 min	79.70±4.26	78.70±9.18	78.73±8.78	0.850	0.295	0.295	0.494
30 min	79.97±4.59	79.43±8.43	79.27±8.35	0.930	0.383	0.347	0.469
35 min	81.46±4.33	79.41±8.15	81.43±7.49	0.435	0.122	0.492	0.163
40 min	79.08±4.46	80.28±6.71	80.31±8.22	0.746	0.222	0.253	0.494
45 min	81.35±4.69	81.73±6.17	81.87±6.88	0.959	0.410	0.389	0.470
50 min	80.68±5.54	82.40±7.47	82.52±5.02	0.580	0.203	0.139	0.474
55 min	80.76±6.11	81.10±5.58	80.88±6.92	0.986	0.431	0.481	0.457
60 min	79.93±3.91	80.93±6.5	81.80±6.53	0.727	0.313	0.195	0.375
65 min	81.63±3.62	80.56±70	82.00±1.58	0.855	0.352	0.416	0.331
70 min	79.80±4.32	81.50±4.36	81.33±7.23	0.864	0.289	0.357	0.485
75 min	80.50±7.78	78.67±2.52	81.00	-	0.356	-	-

The fall in MAP was more in group C followed by group B and comparatively less fall in blood pressure was reported in group A till 18 minutes and was statistically significant among the groups (P<0.005). However, the mean values remained comparable between groups A and B at various time intervals. After 18 minutes MAP stabilized in all the groups and was statistically not significant among the groups (P>0.05) (Table 3).

**Table 3:** Comparison of mean arterial pressure (mmHg) among the study groups

Time (minutes)	Group A (n=30)	Group B (n=30)	Group C (n=30)	P value	P value		
	Mean±SD	Mean±SD	Mean±SD		A vs B	A vs C	B vs C
0 min	98.13±3.41	98.31±3.82	97.60±2.49	0.686	0.425	0.246	0.198
02 min	91.74±3.93	91.67±3.48	88.89±3.55	0.004*	0.468	0.002*	0.002*
04 min	88.33±4.27	88.69±3.83	84.11±7.2	0.002*	0.368	0.004*	0.002*
06 min	86.73±3.81	88.37±4.75	82.90±8.89	0.003*	0.074	0.017*	0.002*
08 min	86.17±6.79	85.87±5.28	81.60±6.91	0.010*	0.425	0.006*	0.005*
10 min	87.52±5.36	87.09±3.2	82.83±6.71	0.001*	0.353	0.002*	0.001*
12 min	88.25±5.77	87.26±6.38	83.59±5.86	0.009*	0.266	0.001*	0.012*
14 min	88.38±4.71	88.52±4.33	84.89±4.55	0.003*	0.451	0.003*	0.001*
16 min	88.80±3.95	89.37±6.33	84.25±7.43	0.003*	0.340	0.002*	0.003*
18 min	88.81±3.59	89.61±8.21	84.38±7.2	0.006*	0.313	0.002*	0.006*
20 min	88.20±4.9	87.91±8.51	86.33±7.5	0.553	0.436	0.129	0.224
25 min	88.22±5.12	87.54±5.98	86.29±5.78	0.409	0.320	0.088	0.206
30 min	89.16±5.54	88.36±6.62	87.89±3.54	0.660	0.308	0.148	0.368
35 min	90.87±5.07	88.66±9.1	89.28±4.89	0.439	0.132	0.116	0.374
40 min	89.01±7.82	87.40±8.74	87.59±8.3	0.742	0.238	0.262	0.467
45 min	90.15±5.89	88.08±8.53	89.13±7.64	0.654	0.179	0.315	0.326
50 min	89.63±5.57	88.72±7.86	90.81±6.66	0.592	0.335	0.275	0.171
55 min	92.12±5.76	85.93±15.83	90.29±4.56	0.202	0.068	0.166	0.155
60 min	94.62±4.8	91.69±7.75	90.89±7.51	0.359	0.123	0.080	0.406
65 min	90.74±3.19	92.48±7.26	91.40±6.4	0.815	0.260	0.399	0.393
70 min	90.00±2.66	93.50±5.21	87.33±7.57	0.307	0.115	0.241	0.127
75 min	90.50±2.59	92.00±12.86	98.33-	-	0.443	-	-

The peripheral oxygen saturation was comparable among the groups at all time periods during the surgery (P-value >0.05).

Characteristics of the subarachnoid blockade

Time to reach peak sensory block level T4 was fastest in group A (09.17±1.34 min), followed by group B (09.33±1.37 min) and slowest in group C (09.37±1.33 min) and was comparable among the groups (p=0.999). The rate of two-segment sensory regression (T4 to T6) was fastest in group B (100.10±6.29 min) than in group A (104.70±5.16 min) and slowest in group C (105.83±3.9 min) and difference was statistically significant (p=0.003*). The rate of sensory block regression to levels T10, T12, and S1 was fastest in group B (68.00±6.23 min, 120.33±4.94 min, 161.27±4.98 min respectively) than in group A (72.27±5.11 min, 124.83±7.22 min, 170.20±6.06 min respectively) and group C (72.13±4.88 min, 126.67±7.47 min, 171.90±5.09 min respectively) and the difference was statistically significant (p=0.001*, 0.001*, 0.001* respectively) (Table 4).

**Table 4:** Comparison of sensory block among the groups

Sensory Block (minutes)	Group A (n=30)	Group B (n=30)	Group C (n=30)	P value	P value		
	Mean±SD	Mean±SD	Mean±SD		A vs B	A vs C	B vs C
Peak Sensory Block T4	9.17±1.34	9.33±1.37	9.37±1.33	0.035	0.318	0.282	0.462
Two Segment Regression T4 to T6	72.27±5.11	68.00±6.23	72.13±4.88	0.029*	0.003*	0.459	0.003*
Sensory Regression to T10	104.70±5.16	100.10±6.29	105.83±3.9	<0.001*	0.002*	0.171	<0.001*
Sensory Regression to T12	124.83±7.22	120.33±4.94	126.67±7.47	<0.001*	0.003*	0.169	<0.001*
Sensory Regression to S1	170.20±6.06	161.27±4.98	171.90±5.09	<0.001*	<0.001*	0.122	<0.001*

The time to onset of motor block (MBS=4) was fastest in group A (7.83±1.05 min) than in group B (7.90±1.12 min), and slowest in group C (8.17±1.05 min) and comparable among the groups (p=0.999). The regression of motor block (MBS=0) was faster in group B (154.73±4.73 min) than in group A (161.30±5.81 min), and slowest in group C (163.07±4.65 min) and the difference was statistically significant (p=0.001*) (Table 5).

**Table 5:** Comparison of motor block among the groups

Motor Block (minutes)	Group A (n=30)	Group B (n=30)	Group C (n=30)	P value	p-value		
	Mean±SD	Mean±SD	Mean±SD		A vs B	A vs C	B vs C
Time to Modified Bromage Scale = 4	7.83±1.05	7.90±1.12	8.17±1.05	0.436	0.407	0.113	0.174
Time to Modified Bromage Scale = 3	114.07±2.42	112.10±2.29	114.90±4.36	0.003*	0.001*	0.182	0.001*
Time to Modified Bromage Scale = 0	161.30±5.81	154.73±4.73	163.07±4.65	0.001*	0.001*	0.099	0.001*

Adverse effects

Nausea was reported in 3.33%, 3.33%, and 36.67% in groups A, B, and C, respectively and the mean difference was significant among the groups (p=0.001*). Bradycardia was reported in 6.67% and 10.00% in groups B and C, respectively and no case was reported in group A and the mean difference was significant among the groups (p=0.021*). Shivering was reported in 6.67%, 3.33%, and 33.33% in groups A, B, and C, respectively and the mean difference was significant among the groups (p=0.001*). Ephedrine used was reported in 6.67%, 13.33%, and 36.67% of groups A, B, and C, respectively and the mean difference was significant among the groups (p=0.003*). The pain was reported in 6.67%, 10.00%, and 10.00% in groups A, B, and C, respectively and comparable among the groups (p=0.856) (Table 6).

**Table 6:** Comparison of adverse effects among the groups

Adverse Effects	Group A (n=30)		Group B (n=30)		Group C (n=30)		P value
	n	%	n	%	n	%	
Nausea	1	3.33%	1	3.33%	11	36.67%	0.001*
Bradycardia	0	0.00%	2	6.67%	3	10.00%	0.021*
Shivering	2	6.67%	1	3.33%	10	33.33%	0.001*
Pain	2	6.67%	3	10.00%	3	10.00%	0.856
Ephedrine Use	2	6.67%	4	13.33%	11	36.67%	0.003*

## Discussion

Spinal anaesthesia is a widely used anaesthetic technique for both elective and emergency surgeries. Spinal anaesthesia also prevents the possible excess systemic blood levels of local anaesthetics which can lead to life-threatening cardiovascular or neurological manifestations [12]. The serotonin receptors are abundantly present in the superficial laminae and substantia gelatinosa of the spinal cord [13]. 5-HT3 receptors are extensively distributed both peripherally, on abdominal vagal nerve terminals and centrally in the chemoreceptor trigger zone of the area postrema and the nucleus tractus solitarius [14]. Although the spinal serotonergic mechanisms in pain modulation are complex, several studies have confirmed the role of 5-HT3 receptors in antinociception. The antiemetic action of the 5-HT3 antagonists is due to simultaneous effects at both central and peripheral 5-HT3 receptor sites. Due to the wide dose range of tolerance, FDA-approved four 5-HT3 antagonists (ondansetron, granisetron, dolasetron, palonosetron) to combat nausea and vomiting. Granisetron, in contrast to ondansetron (acts on mixed receptors) strongly and selectively binds to the 5-HT3 receptors with minimal or no affinity for other 5-HT serotonin receptors, dopaminergic, adrenergic, histaminic, and opioid receptors. Hence granisetron has faster regression of sensory effect in comparison to ondansetron [6].

In the present study, the mean heart rate showed better stability in the ondansetron group as compared to the granisetron group from the baseline value. However, the difference was statistically not significant between the groups (P>0.05). Our results were inconsistent with the study conducted by Kumar A et al. [15] who found that the mean heart rate was more stable in the ondansetron group as compared to the granisetron group. The difference was statistically not significant between the groups (P>0.05). Another study was conducted by Khalifa [14]. Ahmed Abdelbaset Mostafa et al. [16] also reported similar results in their study. In the present study, MAP remained more stable in the ondansetron group compared to the granisetron group but the mean difference was statistically not significant between the groups (P>0.005). Ahmed Abdelbaset Mostafa et al. [16] conducted a randomized double-blind study and observed that hemodynamic variables like SBP, DBP, and MAP showed more stability in the ondansetron group compared to the granisetron group. However, the difference was statistically not significant (P>0.05). This was also in concordance with the study conducted by Rashad et al. [17], Khalifa et al. [14], Ray D et al. [18], and Kumawat et al. [19].

In our study, the mean time to reach peak sensory block level T4 was comparable among the groups (P>0.05). The rate of sensory block regression to two segments, T4 to T6 and thereafter to T10, T12, and S1 was faster in the granisetron group than the ondansetron group and the difference was statistically significant (P<0.05). The above findings were also supported by Rashad et al. [17] who observed that rate of sensory block regression to two segments was faster in the granisetron group compared to the ondansetron group and the difference was statistically significant (P<0.05). Kumar A et al. [15] and Mostafa Abu Bakr Megahed et al. [20] also observed similar results in support of our study. In our study, the duration of motor blockade (MBS=3 and MBS=0) was maximum for group A compared to group B and the difference was statistically significant (P<0.05). In a randomized study, Ray D et al. [18] reported that motor blockade time was maximum in patients premedicated with intravenous ondansetron than granisetron group (P<0.001). The results were consistent with our study.

In our study, complications like bradycardia, shivering, nausea, vomiting, and ephedrine used were more in the normal saline group as compared to the ondansetron group and granisetron group and the difference was statistically significant among the groups (P<0.05). The episodes of pain were comparable among all the groups (p>0.05). Puri A et al. [21] observed that the incidence of shivering, nausea, vomiting and use of ephedrine were more in a normal saline group compared to the ondansetron group and the granisetron group. Similar observations were also reported in our study. Another study done by Mostafa Abu Bakr Megahed et al. [20] reported that the incidence of bradycardia, shivering, nausea, and vomiting was less in the group ondansetron and granisetron group as compared to the normal saline group. Many other studies like Makker et al. [22] and Kumar A et al. [15] also supported our study.

Limitation

The sample size was small, so the findings with regard to the incidence of side effects or the hemodynamic changes as the primary outcome variable cannot be commented on. A low dose of study drugs was another limitation of our study. A fixed dose of 0.5% bupivacaine 2mL was used for the subarachnoid block as another limitation.

## Conclusions

From the present study, it can be concluded that ondansetron provides better hemodynamic stability, earlier onset of the sensory and motor block as well as prolonged duration of sensory and motor blocks, thus duration of analgesia is longer as compared to patients receiving granisetron. However, premedication with low doses of intravenous ondansetron 4mg and granisetron 1mg effectively reduced the incidence of nausea and vomiting, shivering, hypotension, and bradycardia and markedly reduced usage of vasopressor in patients undergoing surgery under spinal anaesthesia.
